# Dietary *Weizmannia faecalis* DSM 32016 probiotic supplementation reduces *Salmonella* colonization and supports growth performance in broilers challenged with *Salmonella enterica* serovars

**DOI:** 10.1016/j.psj.2026.107881

**Published:** 2026-10-05

**Authors:** Lydia Zeibich, Alexandra Schlagheck, Ali R. Al Sulaiman, Max Ingberman, Ala E. Abudabos, Breno Castello Branco Beirão

**Affiliations:** a Biochem Zusatzstoffe Handels- und Produktionsgesellschaft mbH, Lohne, Germany; b Environmental Protection Technologies Institute, Sustainability and Environment Sector, King Abdulaziz City for Science and Technology, P.O. Box 6086, Riyadh, 11442, Saudi Arabia; c Imunova Análises Biológicas LTDA, Curitiba, Brazil; d Department of Food and Animal Sciences, College of Agriculture, Tennessee State University, Nashville, TN, 37209, USA; e Federal University of Paraná (UFPR), Curitiba, Brazil

**Keywords:** *Weizmannia faecalis*, Probiotic, Broiler, *Salmonella enterica*, Growth performance

## Abstract

*Salmonella* colonization in broilers remains a major challenge for poultry production and food safety, increasing the need for non-antibiotic nutritional strategies that support intestinal health and pathogen control. This research evaluated the efficacy of *Weizmannia faecalis* DSM 32016 (WF-32016) as a dietary probiotic supplement in reducing *Salmonella* colonization and supporting intestinal health and growth performance in broilers under experimental challenge conditions. Two complementary experiments were conducted using Ross 308 broilers. In Study I, 216 birds were allocated to six treatments representing the combination of two *Salmonella enterica* serovar Typhimurium challenge conditions (non-challenged and challenged) and three dietary regimens: an unsupplemented control, WF-32016 at 400 g/t during the starter phase and 200 g/t during the finisher phase (WF 400/200), or WF-32016 at 400 g/t during both phases (WF 400/400). Growth performance was evaluated throughout the experimental period, and cecal microbial populations, serum malondialdehyde (MDA) concentration, and ileal histomorphology were assessed on day 35. In Study II, 108 birds were allocated to three treatments: non-challenged control, *Salmonella enterica* serovar Minnesota-challenged control, and *Salmonella* Minnesota-challenged birds receiving WF-32016 at 400 g/t during the starter phase and 200 g/t during the finisher phase (WF 400/200). Cecal *Salmonella* colonization and intestinal permeability were assessed on days 7 and 28. In Study I, *Salmonella* challenge impaired daily weight gain, feed conversion ratio, and production efficiency factor, particularly during the finisher and overall periods. WF-32016 supplementation improved performance in challenged broilers, with the WF 400/400 regimen producing the most consistent response. Cecal *Salmonella* enumerations were numerically reduced by WF-32016 in challenged birds, with the greatest reduction observed in the WF 400/400 group. Supplementation also lowered serum MDA concentrations and numerically increased cecal *Lactobacillus* populations, while ileal morphology was not adversely affected. In Study II, WF-32016 significantly reduced cecal *Salmonella* colonization on both days 7 and 28, although intestinal permeability did not differ among treatments. Overall, dietary WF-32016 reduced *Salmonella* colonization and supported broiler performance under enteric challenge conditions, indicating its potential as a probiotic strategy for improving poultry health and productivity.

## Introduction

Poultry ranks among the most widely consumed animal proteins globally, and its production and trade are pivotal in meeting the rising demand for animal-based food products (Govoni et al., 2021). Nevertheless, foodborne pathogens, particularly *Salmonella*, pose substantial challenges to both public health and the poultry industry (Nair and Kollanoor Johny, 2019; Zizza et al., 2024). Contamination of poultry meat with *Salmonella* remains a persistent concern, especially in regions that serve as major exporters to global markets (Alikhan et al., 2022; Thames et al., 2022; Queiroz et al., 2023). Beyond its direct threat to food safety, the presence of *Salmonella* restricts trade opportunities, as stringent international regulations enforce zero-tolerance policies for *Salmonella*-positive shipments, particularly for serovars Typhimurium, Enteritidis, and Infantis (Tykałowski and Koncicki, 2024). This regulatory landscape places considerable pressure on poultry-exporting nations to implement robust mitigation strategies. Countries such as Brazil, the world's largest poultry exporter, face compounding challenges related to *Salmonella* prevalence, driven by climatic conditions, biosecurity limitations, intensive production systems, and the pathogen's natural persistence in poultry environments (Alikhan et al., 2022).

*Salmonella* is a genus encompassing over 2,500 serotypes, many of which are implicated in human foodborne illness (Chattaway et al., 2021; Zizza et al., 2024). *Salmonella enterica* serovar Typhimurium (*S*. Typhimurium) is among the most frequently isolated serotypes worldwide and is associated with significant human infections and economic losses in the poultry sector, alongside *Salmonella enterica* serovar Enteritidis (Nazari Moghadam et al., 2023). *S*. Typhimurium is highly invasive, capable of colonizing the avian intestinal tract and contributing to contamination of both meat and eggs (Dar et al., 2017). In contrast, *Salmonella enterica* serovar Minnesota (*S*. Minnesota) has been increasingly detected in South American poultry production, most notably in Brazil, and is distinguished by its capacity to persist in both the poultry gut and the broader farm environment, rendering it a particularly difficult pathogen to manage (dos Santos et al., 2024).

An emerging strategy for *Salmonella* control involves the combined use of multiple feed additives with direct or indirect antimicrobial properties, including probiotics, prebiotics, organic acids, and essential oils, to generate synergistic effects against the pathogen under field conditions (Naeem and Bourassa, 2024). This integrated approach leverages the complementary modes of action of these additives to reduce pathogen colonization, support gut health, and enhance bird performance. For instance, probiotics modulate the gut microbiome and directly inhibit harmful bacteria, while organic acids lower intestinal pH to create an environment hostile to *Salmonella* (Van Immerseel et al., 2006; Gao et al., 2021).

Probiotics, defined as live microorganisms that confer health benefits when administered in adequate quantities, represent an effective strategy for improving poultry health and productivity (FAO/WHO, 2001). Within integrated pathogen control frameworks, probiotics may help reduce *Salmonella* colonization in the gastrointestinal tract while supporting overall flock health and performance (Tellez et al., 2012). Their application aligns with the global transition toward sustainable, antibiotic-free poultry production and facilitates compliance with international trade standards. Among promising probiotic candidates, *Weizmannia faecalis*, formerly classified as *Bacillus coagulans*, has attracted particular interest. *Weizmannia* species are heat-stable, spore-forming bacteria that are well suited for incorporation into pelleted feeds. They are homofermentative and produce lactic acid efficiently (Payot et al., 1999; Sinnelä et al., 2021; Zhang et al., 2024), a property previously associated primarily with *Lactobacillus*- and *Enterococcus*-based probiotics in animal nutrition. Lactic acid has been linked to improvements in intestinal barrier function, immune modulation, and beneficial microbial shifts (Duncan et al., 2004; Bourriaud et al., 2005; Garrote et al., 2015). Furthermore, lactic acid reduces intestinal pH, thereby creating unfavorable conditions for *Salmonella*, which requires a minimum pH of approximately 6.5 for optimal growth (Chung and Goepfert, 1970). Additional proposed mechanisms include interference with pathogen adhesion, competitive exclusion, enzymatic activity, and modulation of the microbial community (Zhou et al., 2020; Raheem et al., 2021).

In humans, *Weizmannia* supplementation has been associated with alleviation of gastrointestinal conditions such as diarrhea and dysbiosis (Dolin, 2009; Cao et al., 2020; Zhou et al., 2020). In animal production, *Weizmannia*-based probiotics have demonstrated improvements in feed efficiency, productive performance, and gut health in both poultry and weaning piglets (Wu et al., 2018; Bampidis et al., 2020; Parhizkar et al., 2022; Elleithy et al., 2023). However, evidence regarding their effectiveness in controlling *Salmonella* in poultry remains limited. To date, relatively few studies have reported reductions in cecal *Salmonella enterica* serovar Enteritidis colonization in challenged broilers supplemented with *Weizmannia* or *Bacillus coagulans* (Zhen et al., 2018; El Mahdy et al., 2021). Further research is therefore needed to determine whether *Weizmannia* can inhibit a broader spectrum of *Salmonella* serovars and meaningfully contribute to poultry health and food safety. Accordingly, the objective of this work was to evaluate the efficacy of *Weizmannia faecalis* DSM 32016 (WF-32016) in broilers across two complementary experimental models. Study I (*S*. Typhimurium challenge) assessed the effects of WF-32016 on growth performance, cecal *Salmonella* colonization, oxidative stress, intestinal *Lactobacillus* population, and gut morphology under both challenged and non-challenged conditions, while Study II (*S*. Minnesota challenge) was designed as a confirmatory model to evaluate WF-32016 efficacy specifically under pathogen challenge conditions, with a focus on cecal *Salmonella* enumeration and intestinal permeability. Although individual performance, microbiological, and intestinal health measurements used in the present research are established endpoints, the experimental approach was designed to address an unresolved question concerning the consistency of the response to WF-32016 across distinct *Salmonella* serovars and experimental environments.

## Materials and methods

### Ethics statement

The experimental protocol was approved by the Ethics Committee of King Saud University, Riyadh, Saudi Arabia (No. KSU-SE-21-38; approved May 27, 2023) for Study I, and by the Ethics Committee of Imunova Análises Biológicas LTDA, Curitiba, Brazil (No. 2024.0011; approved July 26, 2024) for Study II.

### Study I (Poultry House): experimental design and measured parameters

A total of 216 one-day-old, straight-run Ross 308 broiler chicks were obtained from a commercial hatchery. Upon arrival, chicks were confirmed to be *Salmonella*-free by bacteriological culture of cloacal swabs, following the method previously described (McLendon et al., 2020). Birds were randomly assigned to six treatment groups, with six replicates of six birds per treatment. The treatments represented two *S.* Typhimurium challenge conditions (non-challenged [−] and challenged [+]) combined with three dietary regimens: (1) an unsupplemented control diet, (2) WF-32016 at 400 g/t during the starter phase followed by 200 g/t during the finisher phase (WF 400/200), and (3) WF-32016 at 400 g/t during both the starter and finisher phases (WF 400/400). Accordingly, the six experimental groups were (-) control, (-) WF 400/200, (-) WF 400/400, (+) control, (+) WF 400/200, and (+) WF 400/400 (Table 1).

**Table 1:** Experimental treatment groups by *Salmonella* Typhimurium (*S.* Typhimurium) challenge status and dietary *Weizmannia faecalis* DSM 32016 (WF-32016) inclusion level.

Group ID a	Challenge status b	WF-32016 inclusion level (CFU/kg feed) c
(−) Control	Non-challenged	None
(−) WF 400/200	Non-challenged	Starter: 1.0 × 10^9^; Finisher: 0.5 × 10^9^
(−) WF 400/400	Non-challenged	Starter: 1.0 × 10^9^; Finisher: 1.0 × 10^9^
(+) Control	Challenged	None
(+) WF 400/200	Challenged	Starter: 1.0 × 10^9^; Finisher: 0.5 × 10^9^
(+) WF 400/400	Challenged	Starter: 1.0 × 10^9^; Finisher: 1.0 × 10^9^

a (−), non-challenged; (+), challenged; WF 400/200, WF-32016 supplemented at 400 g/t during the starter phase and 200 g/t during the finisher phase; WF 400/400, WF-32016 supplemented at 400 g/t during both the starter and finisher phases.

b Challenged birds were orally inoculated on day 4 with 1 mL of *S.* Typhimurium suspension at 3 × 10⁹ CFU/mL.

c CFU, colony-forming units.

On day 4 of the trial, birds assigned to the three challenged (+) groups were orally inoculated by gavage with 1 mL per bird of *S*. Typhimurium suspension (MicroBiologics, St. Cloud, MN, USA) at a concentration of 3 × 10⁹ CFU/mL. The required quantity of the probiotic strain WF-32016 (TechnoSpore; Biochem Zusatzstoffe Handels- und Produktionsgesellschaft mbH, Lohne, Germany) was weighed according to the designated inclusion rate for each supplemented treatment and incorporated directly into the respective feed during mixing to achieve homogeneous distribution throughout the complete diet. The WF 400/200 treatment provided estimated WF-32016 concentrations of 1.0 × 10⁹ CFU/kg feed during the starter phase (days 0–21) and 0.5 × 10⁹ CFU/kg feed during the finisher phase (days 22–35), whereas the WF 400/400 treatment provided an estimated WF-32016 concentration of 1.0 × 10⁹ CFU/kg feed during both feeding phases.

The starter and finisher diets, composed primarily of corn and soybean meal, were formulated based on the nutritional recommendations for Ross broilers (Aviagen, 2022). The ingredient composition and calculated and analyzed nutrient contents of the experimental diets are presented in Table 2. For chemical analysis, crude protein content was determined using the combustion method of AOAC 990.03, while calcium and phosphorus concentrations were quantified using the spectrophotometric method of AOAC 968.08 and the photometric method of AOAC 965.17, respectively (AOAC, 2019). Diets were prepared to be isocaloric and isonitrogenous among treatment groups and were offered in mash form. Feed and water were provided *ad libitum* throughout the experiment. Birds were reared in a cage housing system within an environmentally controlled poultry facility under environmental and management conditions consistent with Ross strain guidelines.

**Table 2:** Ingredient and nutrient composition of experimental diets (as-fed basis) used in Studies I and II CE: Table Citation not matching with the original source. Please verify. Following are missing in the current document CE: Table Citation not matching with the original source. Please verify. Following are missing in the current document table2^a^.

Ingredient composition, %	Starter phase	Finisher phase
Yellow corn	51.0	58.0
Soybean meal	40.0	30.5
Wheat bran	3.00	4.18
Choline chloride	0.05	0.00
Corn oil	2.00	4.10
Dicalcium phosphate	1.94	1.60
Ground limestone	0.86	0.80
Salt	0.30	0.30
DL-methionine	0.39	0.24
L-lysine	0.26	0.08
Vitamin–mineral premix b	0.20	0.20
**Calculated composition**		
Metabolizable energy, kcal/kg	3000	3200
Crude protein, %	23.0	19.5
Available phosphorus, %	0.48	0.40
Calcium, %	0.96	0.79
Digestible lysine, %	1.28	1.03
Digestible sulfur amino acids, %	0.95	0.80
Digestible threonine, %	0.86	0.69
**Analyzed composition, %**		
Crude protein	23.2	19.6
Phosphorus	0.49	0.40
Calcium	0.93	0.74

a The experimental diets were supplemented with *Weizmannia faecalis* DSM 32016 (WF-32016) according to treatment assignment. In Study I, WF-32016 was included at 400 g/t during the starter phase and at either 200 or 400 g/t during the finisher phase, whereas in Study II, it was included at 400 g/t during the starter phase and at 200 g/t during the finisher phase. Control treatments did not receive WF-32016 supplementation.

b Vitamin–mineral premix supplied per kilogram of premix: vitamin A, 12,000,000 IU; vitamin D_3_, 5,000,000 IU; vitamin E, 80,000 IU; vitamin K_3_, 3200 mg; vitamin B_1_, 3200 mg; vitamin B_2_, 8600 mg; vitamin B_3_, 65,000 mg; pantothenic acid, 20,000 mg; vitamin B_6_, 4300 mg; biotin, 220 mg; vitamin B_9_, 2200 mg; vitamin B_12_, 17 mg; antioxidants, 50,000 mg; copper, 16,000 mg; iodine, 1250 mg; iron, 20,000 mg; manganese, 120,000 mg; selenium, 300 mg; and zinc, 110,000 mg.

Zootechnical parameters, including live body weight and feed intake, were recorded on a per-phase basis and used to calculate the feed conversion ratio (FCR) and overall trial performance indices. Mortality was recorded throughout the experiment, and FCR was corrected for mortality by accounting for the body weight of birds that died, where applicable. The Production Efficiency Factor (PEF) was calculated using the following formula:

PEF=[Livability(%)×Bodyweight(kg)]/[Age(days)×FCR]×100

At the conclusion of the trial (day 35), one bird was randomly selected from each replicate for sample collection and subsequent evaluation of oxidative stress status, ileal histomorphology, and cecal microbial populations.

To determine serum malondialdehyde (MDA) concentrations as an indicator of oxidative stress, blood samples (3 mL per bird) were collected from the brachial vein. The samples were centrifuged at 3,000×g for 10 min, and the separated serum was stored at −20°C until analysis. Serum MDA concentrations were measured using a thiobarbituric acid reactive substances assay with a commercially available ELISA kit (Cayman Chemical Company, Ann Arbor, MI, USA).

For histomorphological evaluation, roughly 2 cm portions of the distal ileum were obtained. The samples were washed with phosphate-buffered saline and preserved in 10% buffered formalin until subsequent laboratory processing. Sections measuring 5 μm in thickness were then prepared, placed on glass slides, and stained with haematoxylin and eosin (Abdelqader and Al-Fataftah, 2016). For each treatment, at least 50 properly oriented villi were selected to determine villus height and width using image-analysis software (Image-Pro, Media Cybernetics, Inc., Silver Spring, MD, USA). Villus surface area was then estimated using the following equation:

$$\text{Villus}\;\text{surface}\;\text{area}\left(\mu m^{2}\right)=2\pi \times \left[\left(villus\;width,\mu m\right)/2\right]\times villus\;height\left(\mu m\right)$$

For microbial evaluation, 1 g of cecal digesta was obtained and aseptically placed into sterile containers. Each sample was homogenized with sterile saline at a 1:9 (wt/vol), after which serial tenfold dilutions were prepared using sterile saline at a 1:9 (vol/vol). From each dilution, approximately 100 µL was plated in duplicate onto selective agar media (Oxoid, Basingstoke, Hampshire, UK) to quantify the bacterial populations of interest. *Salmonella* enumerations were determined on xylose lysine deoxycholate agar following aerobic incubation at 37°C for 24 h, whereas *Lactobacillus* populations were quantified on de Man, Rogosa and Sharpe agar after anaerobic incubation at 37°C for 48 h. Bacterial counts were reported as log_10_ CFU/g of cecal content.

### Study II (Isolators): experimental design and measured parameters

A total of 108 one-day-old, straight-run Ross 308 broiler chicks were obtained from a commercial hatchery and housed in positive-pressure isolators (Fig. 1). The isolator model was used to minimize background microbial variation and standardize exposure conditions during the challenge period, rather than to simulate a commercial rearing system. Birds were randomly allocated to three experimental groups: (1) non-challenged, unsupplemented control [(−) control]; (2) *S.* Minnesota-challenged, unsupplemented control [(+) control]; and (3) *S.* Minnesota-challenged birds supplemented with WF-32016 at 400 g/t during the starter phase and 200 g/t during the finisher phase [(+) WF 400/200] (Table 3). Each treatment group comprised six replicates, with six birds per replicate.

**Fig. 1:**
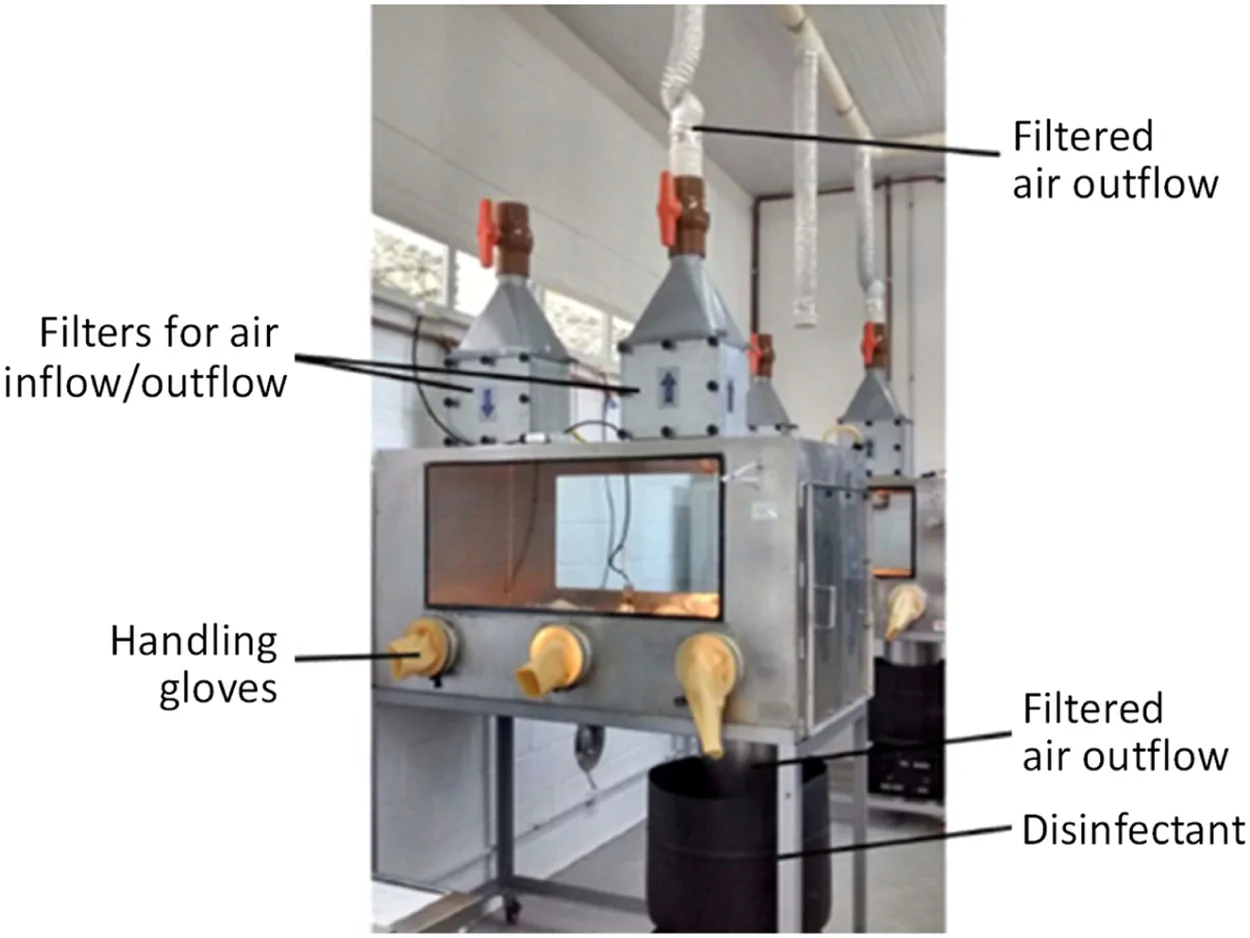
Isolation unit used for broilers challenged with *Salmonella enterica* serovar Minnesota in Study II.

**Table 3:** Experimental treatment groups by *Salmonella* Minnesota (*S.* Minnesota) challenge status and dietary *Weizmannia faecalis* DSM 32016 (WF-32016) inclusion level.

Group ID a	Challenge status b	WF-32016 inclusion level (CFU/kg feed) c
(−) Control	Non-challenged	None
(+) Control	Challenged	None
(+) WF 400/200	Challenged	Starter: 1.0 × 10^9^; Finisher: 0.5 × 10^9^

a (−), non-challenged; (+), challenged; WF 400/200, WF-32016 supplemented at 400 g/t during the starter phase and 200 g/t during the finisher phase.

b Challenged birds were orally inoculated on day 1 with 1 mL of *S.* Minnesota suspension at 1 × 10⁹ CFU/mL.

c CFU, colony-forming units.

On day 1 of the trial, birds in the two challenged (+) treatments were orally inoculated by gavage with 1 mL per bird of S. Minnesota suspension from MicroBiologics at a concentration of 1 × 10⁹ CFU/mL. The (+) WF 400/200 treatment provided estimated WF-32016 concentrations of 1.0 × 10⁹ CFU/kg feed during the starter phase (days 0–15) and 0.5 × 10⁹ CFU/kg feed during the finisher phase (days 16–28).

All birds received starter and finisher diets composed primarily of corn and soybean meal, formulated based on the nutritional recommendations for Ross broilers (Aviagen, 2022), and offered in mash form. The ingredient composition and calculated and analyzed nutrient contents of the experimental diets are presented in Table 2. Feed and water were provided *ad libitum* throughout the trial, and absorbent paper was used as bedding material. Birds were maintained under environmental and management conditions consistent with Ross strain recommendations.

On days 7 and 28, cecal contents were collected from eight randomly selected birds per treatment group to evaluate *Salmonella* cecal colonization, and blood samples were collected from seven randomly selected birds per treatment group to assess intestinal permeability.

*Salmonella* quantification was performed using a methodology based on ISO 6579-1:2017. Samples were weighed and serially diluted in buffered peptone water as the non-selective medium and incubated under standardized conditions. The cultures were subsequently transferred to Rappaport–Vassiliadis soya broth for selective enrichment. Each dilution was then plated onto xylose lysine deoxycholate agar as the selective differential medium, and the qualitative results obtained at each dilution level were used to calculate the most probable number (MPN) value (Blodgett, 2023). Presumptive *Salmonella* colonies were confirmed using a panel of biochemical tests, including indole production, L-tryptophan deaminase activity, sucrose and glucose fermentation, gas production, hydrogen sulfide production, urease activity, lysine decarboxylase activity, and motility assessment. A subset of randomly selected isolates was further confirmed by poly-O agglutination (Grimont and Weill, 2007).

Intestinal permeability was assessed by oral administration of fluorescein isothiocyanate-dextran (FITC-Dextran; molecular weight 3,000–4,000 Da), a non-absorbable fluorescent tracer. Plasma FITC-Dextran concentrations were measured 2.5 h after administration, as intestinal translocation of the marker occurs in proportion to the degree of epithelial barrier disruption. Quantification was performed by comparison with a FITC-Dextran standard curve (Vicuña et al., 2015).

### Statistical analysis

Prior to statistical analysis, data normality was evaluated using the Shapiro–Wilk test, and homogeneity of variance was assessed using Levene’s test. The individual bird was considered the experimental unit, except for performance measures, for which the replicate was considered the experimental unit. Data were analyzed using one-way ANOVA in a completely randomized design, followed by Tukey’s post hoc test for multiple pairwise comparisons, using IBM SPSS Statistics software (version 30; IBM Corp., Armonk, NY, USA). Variables that did not meet the assumptions of normality were analyzed using the Kruskal–Wallis non-parametric test, followed by Dunn’s post hoc test for multiple comparisons. In addition, to evaluate effects that could not be adequately assessed through univariate one-way comparisons alone, multivariate analysis of variance (MANOVA) was performed to assess the main effects of probiotic supplementation and *Salmonella* challenge, as well as their interaction term (Table S1). Differences among groups were considered significant at *P* < 0.05, whereas 0.05 < *P* < 0.10 was interpreted as a statistical trend. Graphs were generated using GraphPad Prism software (version 8.0; GraphPad Software, San Diego, CA, USA).

## Results

### Growth performance

In Study I, dietary supplementation with WF-32016 affected broiler growth performance primarily during the finisher phase and over the overall experimental period (Table 4). During the starter phase, no significant differences were observed among treatment groups for daily feed intake (DFI), daily weight gain (DWG), FCR, or PEF (*P* > 0.05). However, the multivariate analysis indicated a significant main effect of *S*. Typhimurium challenge during the starter phase on DFI, DWG, FCR, and PEF, whereas probiotic supplementation and the *Salmonella* × probiotic interaction were not significant during this phase.

**Table 4:** Effects of dietary supplementation with *Weizmannia faecalis* DSM 32016 (WF-32016) on growth performance of broilers challenged with *Salmonella* Typhimurium (*S.* Typhimurium) during the starter, finisher, and overall experimental periods.

Experimental phase/group	(−) Control b	(−) WF 400/200	(−) WF 400/400	(+) Control	(+) WF 400/200	(+) WF 400/400	*P* value
Starter phase (days 0–21)							
DFI (g) c	47.7 ± 1.6	46.4 ± 3.0	46.9 ± 2.0	44.9 ± 3.7	45.0 ± 1.0	45.6 ± 3.1	0.272
DWG (g)	35.7 ± 2.4	35.6 ± 3.7	35.0 ± 1.9	32.1 ± 2.8	33.3 ± 1.6	33.4 ± 3.1	0.166
FCR (g/g)	1.341 ± 0.044	1.350 ± 0.075	1.339 ± 0.059	1.396 ± 0.047	1.356 ± 0.048	1.373 ± 0.040	0.331
PEF (%)	283 ± 26	271 ± 42	276 ± 18	248 ± 23	259 ± 15	258 ± 27	0.149
Finisher phase (days 22–35)							
DFI (g)	124.1 ± 5.4	126.0 ± 6.0	127.6 ± 5.6	123.9 ± 9.0	126.3 ± 4.4	126.9 ± 2.7	0.820
DWG (g)	71.9^a^ ± 3.0	73.6^a^ ± 3.1	77.4^a^ ± 2.9	66.7^a^ ± 4.3	71.1^a^ ± 3.2	74.4^a^ ± 2.8	<0.001
FCR (g/g)	1.724^a^ ± 0.056	1.717^a^ ± 0.062	1.647^a^ ± 0.052	1.858^a^ ± 0.044	1.756^a^ ± 0.029	1.709^a^ ± 0.040	<0.001
PEF (%)	297^a^ ± 14	297^a^ ± 20	313^a^± 8.0	258^a^ ± 20	284^a^ ± 13	296^a^ ± 17	<0.001
Overall period (days 0–35)							
DFI (g)	78.3 ± 2.7	79.1 ± 2.7	79.1 ± 2.7	76.6 ± 5.5	77.6 ± 2.2	78.1 ± 2.7	0.800
DWG (g)	50.3^a^ ± 1.8	50.0^a^ ± 3.0	51.9^a^ ± 1.7	46.0^a^ ± 3.4	48.9^a^ ± 2.0	49.9^a^ ± 2.3	0.003
FCR (g/g)	1.560^a^ ± 0.042	1.564^a^ ± 0.052	1.522^a^ ± 0.056	1.664^a^ ± 0.034	1.592^a^ ± 0.033	1.573^a^ ± 0.031	<0.001
PEF (%)	328^a^ ± 17	326^a^ ± 25	338^a^ ± 8.0	288^a^ ± 24	313^a^ ± 15	322^a^ ± 18	<0.001
Mortality (%)	0.00^a^	0.00^a^	0.00^a^	16.6^a^ ± 1.19	11.1^a^ ± 0.91	8.3^a^ ± 0.78	< 0.01

a Means within the same row bearing different superscript letters differ significantly (*P* < 0.05). Data are presented as means with standard errors based on six replicates per treatment.

b (−), non-challenged; (+), challenged with *S.* Typhimurium; WF 400/200, WF-32016 supplemented at 400 g/t during the starter phase and 200 g/t during the finisher phase; WF 400/400, WF-32016 supplemented at 400 g/t during both the starter and finisher phases.

c DFI, daily feed intake; DWG, daily weight gain; FCR, feed conversion ratio; PEF, production efficiency factor.

During the finisher phase, DFI did not differ among groups (*P* > 0.05). In contrast, DWG, FCR, and PEF were significantly affected by treatment (*P* < 0.001). Challenged control birds showed the lowest DWG (66.7 g), the highest FCR (1.858 g:g), and the lowest PEF (258 %), indicating impaired performance following *Salmonella* challenge. Supplementation with WF-32016 improved these parameters in challenged birds, particularly at the WF 400/400 dosage, which increased DWG to 74.4 g, reduced FCR to 1.709 g:g, and increased PEF to 296 %. Among non-challenged birds, WF 400/400 produced the highest finisher DWG (77.4 g) and PEF (313 %), together with the lowest FCR (1.647 g:g).

Over the overall period, DFI was not significantly different among groups (*P* > 0.05). However, DWG, FCR, and PEF differed significantly among treatments (*P* = 0.003, *P* < 0.001, and *P* < 0.001, respectively). Challenged control birds had the lowest overall DWG (46.0 g), poorest FCR (1.664 g:g), and lowest PEF (288 %). Dietary WF-32016 improved overall performance in challenged birds, with WF 400/400 increasing DWG to 49.9 g, improving FCR to 1.573 g:g, and increasing PEF to 322 %. The multivariate analysis further confirmed significant main effects of *Salmonella* challenge and probiotic supplementation on DWG, FCR, and PEF during the finisher phase and overall period. Significant *Salmonella* × probiotic interactions were also detected for FCR during the finisher phase and overall period, and for PEF during the finisher phase. No mortality was recorded in the non-challenged groups, whereas challenged birds showed the highest mortality in the control group, with significantly lower rates observed in both supplemented groups.

### Cecal salmonella colonization

Cecal *Salmonella* enumerations were affected by challenge status and WF-32016 supplementation (Fig. 2). In Study I (Fig. 2A), *Salmonella* was not detected in non-challenged control or non-challenged WF-supplemented birds at day 35. Challenged control broilers had the highest cecal load of *Salmonella*, reaching approximately 6.21 log_10_ CFU/g. Supplementation with WF-32016 numerically reduced *Salmonella* enumeration in challenged birds to approximately 4.63 and 3.97 log_10_ CFU/g in the WF 400/200 and WF 400/400 groups, respectively. Although supplemented challenged groups were intermediate, the WF 400/400 treatment produced an approximate 2.24-log reduction relative to the challenged control group.

**Fig. 2:**
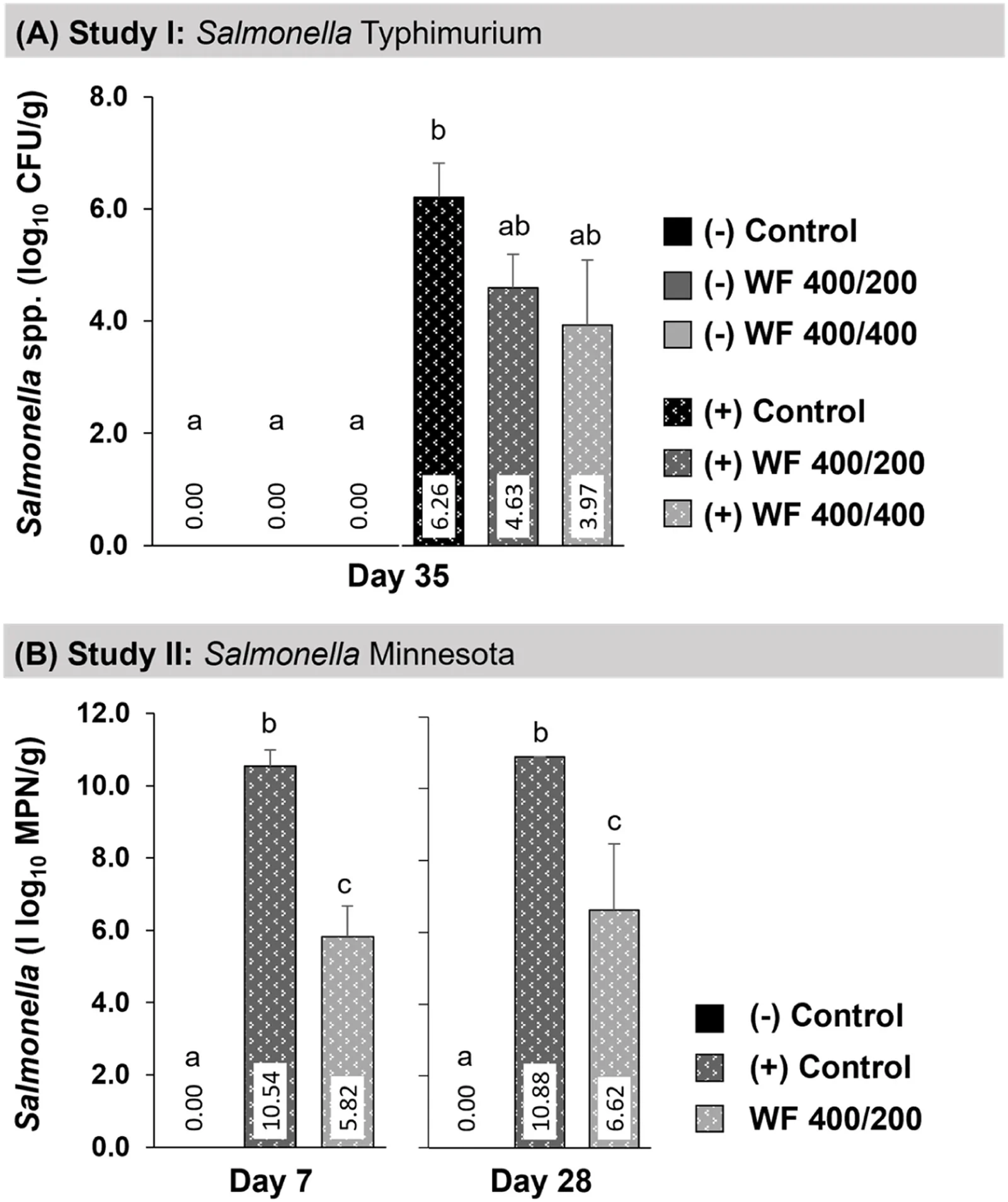
Effect of dietary supplementation with the probiotic *Weizmannia faecalis* DSM 32016 (WF-32016) on cecal *Salmonella* enumerations in broilers challenged with *Salmonella* Typhimurium (A) or *Salmonella* Minnesota (B). Treatments included non-challenged control broilers (−), challenged control broilers (+), broilers supplemented with WF-32016 at 400 g/t during the starter phase and 200 g/t during the finisher phase (WF 400/200), and broilers supplemented with WF-32016 at 400 g/t during both starter and finisher phases (WF 400/400). Results are presented as means with standard errors (*n* = 6 for Study I and *n* = 8 for Study II). Bars with different superscript letters differ significantly (*P* < 0.05).

In Study II (Fig. 2B), *Salmonella* colonization was significantly reduced by WF-32016 supplementation at both sampling points. On day 7, challenged control birds had 10.54 log_10_ MPN/g cecal *Salmonella*, whereas challenged birds receiving WF 400/200 had significantly lower counts of 5.82 log_10_ MPN/g. A similar response was observed on day 28, with *Salmonella* enumerations of 10.88 and 6.62 log_10_ MPN/g in challenged control and challenged WF 400/200 birds, respectively. *Salmonella* was not detected in non-challenged control birds at either time point.

### MDA concentration, lactobacillus population, and intestinal permeability

In Study I, serum MDA concentrations were affected by treatment at day 35 (Fig. 3A). Challenged control birds showed the highest MDA concentration (7.28 µg/g), indicating increased oxidative stress following *S*. Typhimurium challenge. Supplementation with WF-32016 reduced MDA concentrations in challenged birds, with values of 6.93 µg/g in the WF 400/200 group and 5.23 µg/g in the WF 400/400 group. The WF 400/400 challenged group showed MDA concentrations comparable to non-challenged birds, suggesting that the higher probiotic inclusion level attenuated the oxidative stress response associated with *Salmonella* challenge.

**Fig. 3:**
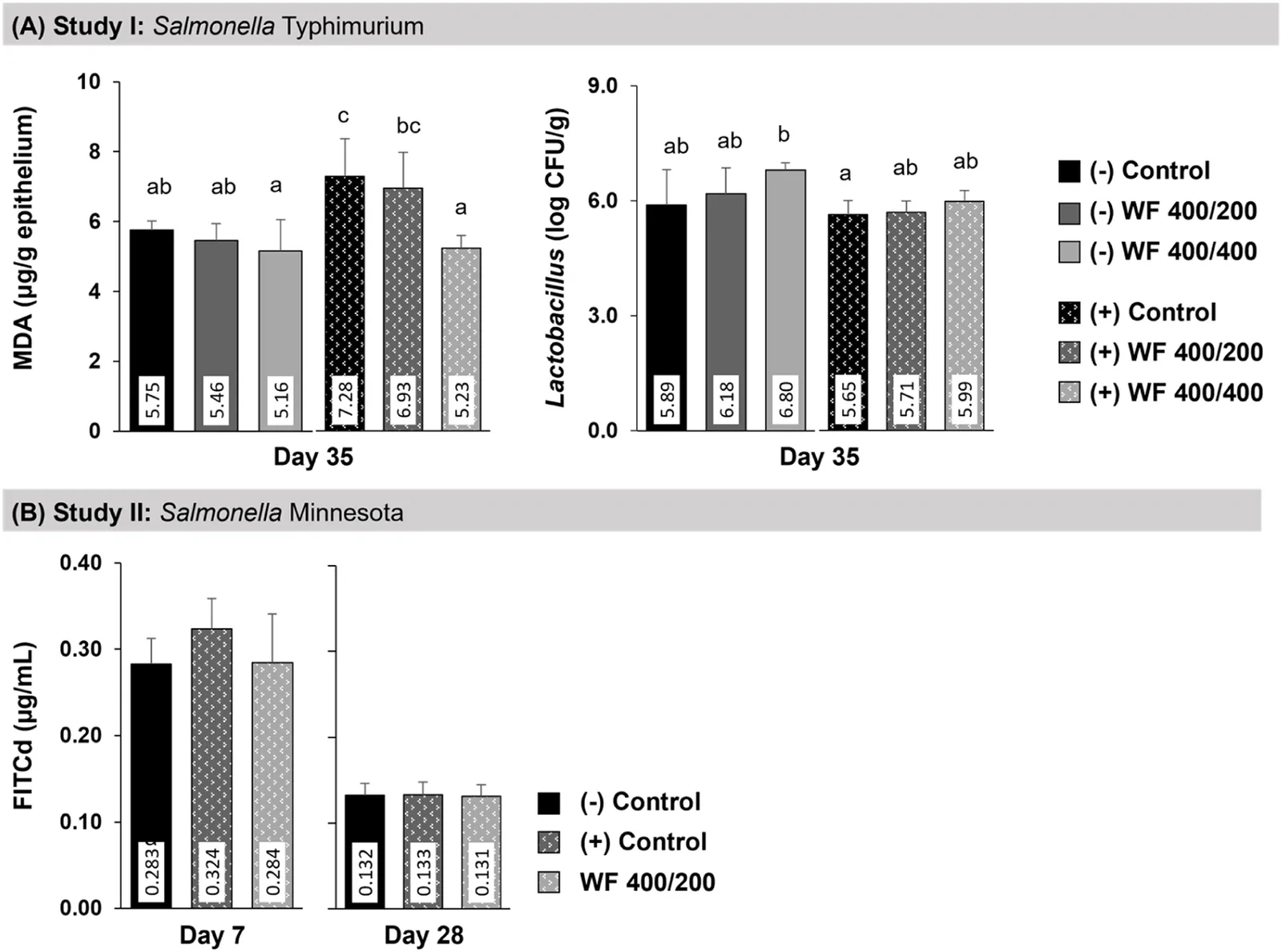
Effect of dietary supplementation with the probiotic *Weizmannia faecalis* DSM 32016 (WF-32016) on malondialdehyde (MDA) concentrations and *Lactobacillus* populations in broilers challenged with *Salmonella* Typhimurium (A), and on intestinal permeability, measured using fluorescein isothiocyanate dextran (FITC-d), in broilers challenged with *Salmonella* Minnesota (B). Treatments included non-challenged control broilers (−), challenged control broilers (+), broilers supplemented with WF-32016 at 400 g/t during the starter phase and 200 g/t during the finisher phase (WF 400/200), and broilers supplemented with WF-32016 at 400 g/t during both starter and finisher phases (WF 400/400). Results are presented as means with standard errors (*n* = 6 for Study I and *n* = 7 for Study II). Bars with different superscript letters differ significantly (*P* < 0.05).

Cecal *Lactobacillus* populations in Study I ranged from 5.65 to 6.80 log_10_ CFU/g (Fig. 3A). The highest value was observed in the non-challenged WF 400/400 group (6.80 CFU/g), while the challenged control group had the lowest value (5.65 CFU/g). Supplemented challenged birds showed intermediate *Lactobacillus* populations, with 5.71 CFU/g in the WF 400/200 group and 5.99 CFU/g in the WF 400/400 group.

In Study II, intestinal permeability, assessed by plasma FITC-dextran concentration, did not differ markedly among treatments at either day 7 or day 28 (*P* > 0.05; Fig. 3B). On day 7, FITC-dextran concentrations were 0.2831 µg/mL in non-challenged controls, 0.324 µg/mL in challenged controls, and 0.284 µg/mL in challenged WF 400/200 birds. On day 28, values were lower across all groups and remained similar among treatments, with concentrations of 0.132, 0.133, and 0.131 µg/mL in the non-challenged control, challenged control, and challenged WF 400/200 groups, respectively.

### Intestinal histomorphology

Ileal histomorphology results from Study I are shown in Fig. 4. No significant treatment-related differences were observed in villus height, villus width, or villus surface area at day 35 (*P* > 0.05). Villus height ranged from 578.1 to 621.3 μm, villus width ranged from 67.2 to 82.2 μm, and villus surface area ranged from 0.122 to 0.159 μm² across treatment groups. Although challenged control birds showed numerically lower villus height and villus surface area than several non-challenged and WF-32016-supplemented groups, these differences were not statistically significant (*P* > 0.05). Overall, WF-32016 supplementation did not adversely affect ileal morphology and numerically supported villus measurements in challenged broilers.

**Fig. 4:**
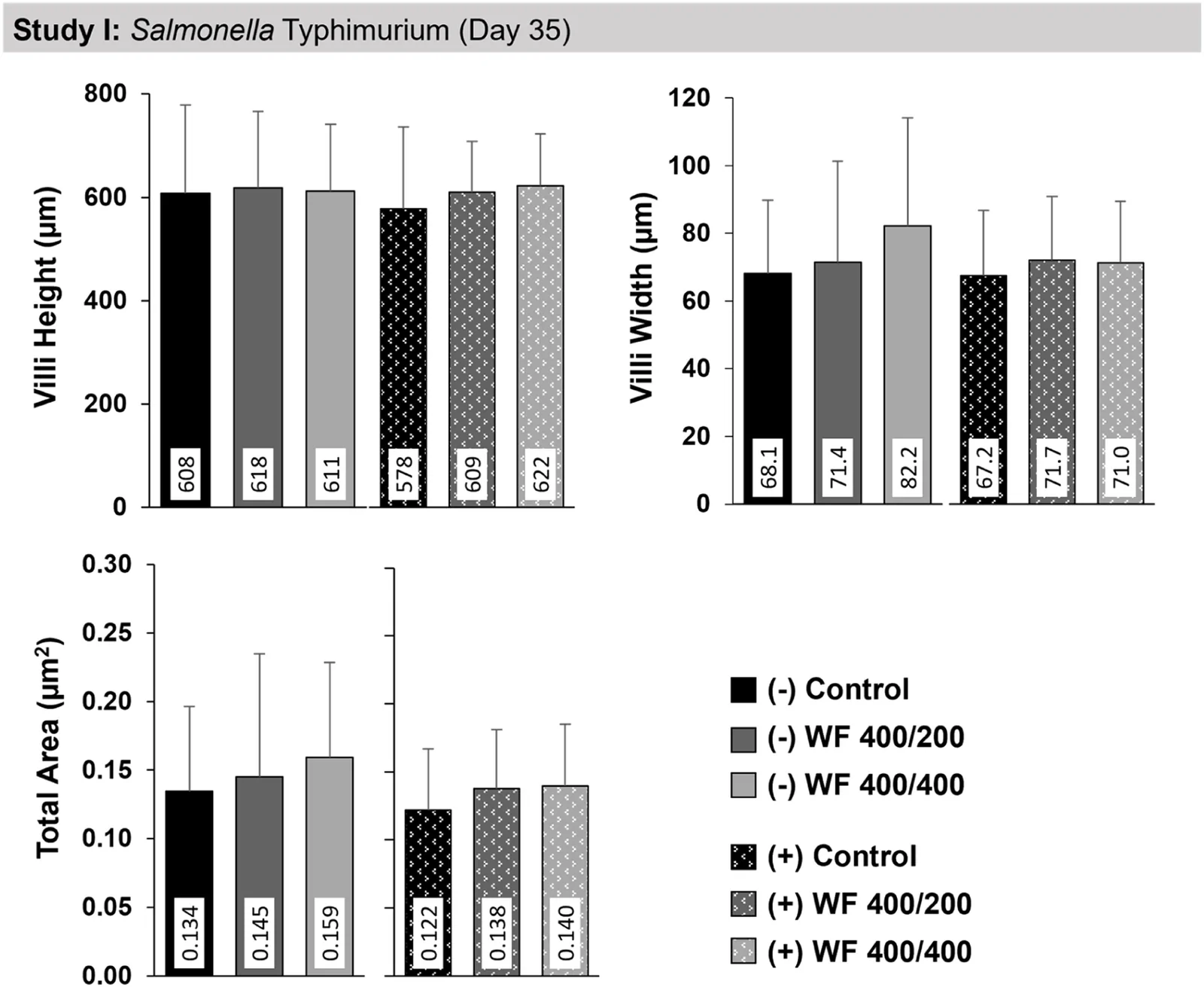
Effect of dietary supplementation with the probiotic *Weizmannia faecalis* DSM 32016 (WF-32016) on Ileal histomorphology in broilers challenged with *Salmonella* Typhimurium. Treatments included non-challenged control broilers (−), challenged control broilers (+), broilers supplemented with WF-32016 at 400 g/t during the starter phase and 200 g/t during the finisher phase (WF 400/200), and broilers supplemented with WF-32016 at 400 g/t during both starter and finisher phases (WF 400/400). Results are presented as means with standard errors (*n* = 6).

## Discussion

The present research evaluated the effects of dietary supplementation with WF-32016 in broilers exposed to two *Salmonella enterica* challenge models. Across the two experiments, WF-32016 reduced cecal *Salmonella* colonization and supported productive performance under challenge conditions. The response was particularly evident in broilers challenged with *S.* Minnesota, in which supplementation significantly reduced cecal *Salmonella* enumerations at both early and late sampling points. In the *S.* Typhimurium model, the probiotic also improved DWG, FCR, and PEF, especially when the higher supplementation level was maintained throughout both feeding phases. These findings indicate that WF-32016 may provide benefits extending beyond pathogen control and may help preserve productive efficiency during enteric challenge.

The impaired growth observed in challenged control birds confirms the biological impact of the S. Typhimurium challenge. Although DFI was not significantly altered during the finisher or overall periods, challenged control birds had lower DWG, poorer FCR, and a lower PEF. This pattern suggests that the challenge reduced the efficiency with which consumed nutrients were converted into body mass rather than merely suppressing feed consumption. Enteric infection can increase nutrient expenditure associated with immune activation, epithelial turnover, tissue repair, and oxidative defense, thereby diverting nutrients away from growth (Naeem et al., 2024). In addition, disruption of the intestinal microbial ecosystem may impair digestion and nutrient utilization even when gross intestinal morphology remains relatively unchanged (Khasanah et al., 2024). WF-32016 supplementation partly counteracted these performance losses. In challenged birds, both supplementation regimens improved finisher and overall performance relative to the challenged control, with the WF 400/400 regimen producing the most consistent response. During the finisher phase, maintaining the higher inclusion rate increased DWG and PEF while reducing FCR. These findings are consistent with Kim et al. (2025), who reported that supplementation with illite combined with *Clostridium butyricum* and *Bacillus subtilis* increased body weight gain and reduced FCR in S. Typhimurium-challenged broilers. The present results also agree with Yin et al. (2024), who found that *Lactiplantibacillus plantarum* GX17 mitigated the reduction in body weight associated with *S. Typhimurium* infection in yellow-feathered broilers. The significant interaction between challenge status and probiotic supplementation detected for FCR further suggest that the effect of WF-32016 depended, at least partly, on the physiological status of the birds. In practical terms, the probiotic appeared to provide greater benefit when birds were subjected to enteric stress than under relatively favorable non-challenged conditions. This is consistent with the concept that probiotic responses are often more apparent when intestinal homeostasis has been disrupted by pathogens, environmental stressors, or other production challenges (Hashemitabar and Hosseinian, 2024).

The absence of significant treatment differences during the starter phase may reflect the time required for probiotic-mediated changes in the intestinal ecosystem to become functionally relevant. Young chicks undergo rapid microbial succession during the first weeks after hatch, and the intestinal microbiota may not yet be sufficiently established for measurable competitive-exclusion effects to be expressed (Lee et al., 2023; Marcato et al., 2024). Furthermore, the performance consequences of the challenge may have accumulated over time and become more pronounced during the finisher period, when nutrient intake and growth rates were greater. The stronger response later in the production cycle therefore does not necessarily indicate a lack of early biological activity, but may instead reflect progressive effects on microbial stability, pathogen pressure, nutrient utilization, and host resilience (Idowu et al., 2025). However, these performance findings should be interpreted with appropriate caution, as the growth performance measurements were based on six independent replicates per treatment. Accordingly, the magnitude of these responses should be considered within the context of the controlled experimental conditions of the present study, and confirmation in further studies with greater replication and under commercial production conditions is warranted.

The reduction in cecal *Salmonella* colonization represents a central finding of the present research. In Study I, challenged birds receiving WF-32016 had numerically lower *Salmonella* enumerations than challenged controls, with the greatest numerical reduction observed in the WF 400/400 group. The cecal *Lactobacillus* results provide limited additional support for a broader microbiota-mediated response. The challenged control group had the lowest numerical *Lactobacillus* population, whereas the highest population was observed in non-challenged birds receiving WF 400/400. Supplemented challenged birds showed intermediate values, with the higher inclusion regimen producing a numerically greater *Lactobacillus* population than the lower regimen. Although these differences were not consistently significant, the overall pattern indicates that WF-32016 did not adversely affect beneficial bacterial populations and may have helped maintain a more favorable cecal microbial environment during *Salmonella* challenge.

A clearer antimicrobial response was observed in Study II, in which WF-32016 significantly reduced *Salmonella* colonization at both sampling ages. This effect was evident by day 7 and persisted through day 28, suggesting that supplementation interfered with both the early establishment and longer-term persistence of *Salmonella* in the ceca. These findings are consistent with Lou et al. (2024), who reported that administration of *Lacticaseibacillus rhamnosus* SN21-1 or *Lactiplantibacillus plantarum* SN21-2 produced a progressive reduction in fecal *S. Typhimurium* enumerations in challenged broilers. This progressive decline supports the present observation that continued probiotic supplementation can restrict the persistence of *Salmonella* after initial colonization. A comparable early antimicrobial response was observed by Khurajog et al. (2026) in S. Typhimurium-challenged broiler chicks receiving a commercial *Clostridium butyricum*-based probiotic. Competitive exclusion is one plausible mechanism underlying this response. WF-32016 may limit pathogen colonization by competing for nutrients or epithelial attachment sites, modifying the physicochemical conditions of the cecal environment, or producing metabolites with inhibitory activity against *Salmonella* (Maurer et al., 2024; Shanmugasundaram et al., 2025). Nevertheless, these mechanisms were not directly evaluated in the present study. Future studies incorporating comprehensive microbiome analysis are therefore warranted to determine whether changes in microbial community structure, diversity, or metabolic activity mediate the observed reduction in *Salmonella* colonization. An additional limitation of the present study is that WF-32016 was not specifically recovered or quantified from intestinal or cecal contents. Therefore, survival of the administered strain during gastrointestinal passage and its persistence or colonization in the gut were not directly demonstrated. Accordingly, the observed reductions in *Salmonella* colonization cannot be attributed specifically to direct colonization or persistence of WF-32016 and may also involve transient probiotic activity or indirect modulation of the resident intestinal microbiota and host environment. Future studies employing strain-specific culture or molecular tracking of WF-32016 are warranted to clarify these mechanisms.

The serum MDA results provide further evidence that WF-32016 enhanced physiological resilience during S. Typhimurium challenge. Challenged control birds exhibited the highest serum MDA concentration, consistent with increased lipid peroxidation and oxidative stress. In contrast, the lower MDA concentrations observed in supplemented challenged birds, particularly those receiving the WF 400/400 regimen, indicate attenuation of the infection-induced oxidative response. This finding is consistent with Abdel-Raheem et al. (2024), who reported that conventional and nano-formulated multistrain probiotics containing *Bacillus subtilis, Lactobacillus plantarum*, and *Bifidobacterium bifidum* reduced MDA and hydrogen peroxide concentrations while increasing glutathione peroxidase and catalase activities in S. Typhimurium-challenged broilers. Similarly, Chen et al. (2024) found that dietary supplementation with *Bacillus subtilis* KG109 significantly reduced serum MDA concentrations and increased total antioxidant capacity, superoxide dismutase activity, and glutathione peroxidase activity in broilers, with responses varying in a dose-dependent manner. This dose-related effect is consistent with the greater reduction in MDA observed under the WF 400/400 regimen in the present study, suggesting that maintaining an adequate probiotic concentration may be important for achieving optimal antioxidant efficacy. Enteric infection and the associated immune response can increase reactive oxygen species production, resulting in cellular lipid damage and a greater metabolic cost of maintaining antioxidant defenses. By reducing pathogen burden, limiting intestinal disruption, and modulating host antioxidant systems, probiotic supplementation may mitigate oxidative damage and preserve nutrients for growth (Liu et al., 2023; Zhang et al., 2024). The association between lower serum MDA concentrations and improved growth performance in the WF 400/400 group supports this interpretation.

The biological basis of the response to WF-32016 may involve several complementary mechanisms. WF-32016 is a spore-forming, lactic-acid-producing strain, characteristics that support survival during feed processing and may allow metabolic activity after ingestion (Elleithy et al., 2023). Lactic acid production can reduce intestinal pH and create conditions less favorable for *Salmonella* growth, while competition for nutrients and epithelial attachment sites may further restrict pathogen establishment (Naeem and Bourassa, 2024; Maurer et al., 2024). Indirect modulation of the resident microbiota and host antioxidant responses may also contribute (Idowu et al., 2025), although these mechanisms were not directly measured in the present experiments and therefore remain proposed explanations for the observed responses. The two challenge organisms also represent distinct biological pressures. *S.* Typhimurium is an invasive serovar capable of intestinal colonization and systemic interaction with the host (Wang et al., 2024), which is consistent with the observed effects on growth performance and oxidative stress in Study I. In contrast, *S.* Minnesota is particularly relevant to poultry production because of its capacity to persist within the poultry intestinal tract and production environment (dos Santos et al., 2024). Reduction of *Salmonella* colonization in Study II therefore primarily demonstrates an effect on intestinal carriage under the conditions of that model. Importantly, the magnitude of the responses between Studies I and II should not be directly compared. The studies were intentionally designed as complementary models and differed in serovar, challenge conditions, housing environment, sampling schedule, and measured endpoints. Accordingly, the value of the two models lies in demonstrating the response to WF-32016 under distinct experimental conditions rather than providing a basis for ranking its efficacy between *Salmonella* serovars.

## Conclusion

Dietary supplementation with WF-32016 was associated with reduced cecal *Salmonella* colonization and selected improvements in broiler performance and physiological responses under experimental challenge conditions. In the *S.* Typhimurium model, reductions in cecal *Salmonella* were numerical, whereas improvements in growth performance and lower serum MDA concentrations were observed, particularly when the higher supplementation level was maintained throughout the trial. In the complementary *S.* Minnesota model, WF-32016 significantly reduced cecal *Salmonella* colonization at both sampling ages, although intestinal permeability was not significantly affected. Ileal histomorphology was also unchanged in Study I. Because the two studies differed in experimental conditions, they should be interpreted as complementary models rather than as a direct comparison of probiotic efficacy between serovars. Overall, these findings support further evaluation of WF-32016 as part of non-antibiotic strategies for managing *Salmonella* challenge in broilers. Further studies with greater replication and under commercial field conditions are warranted to confirm the magnitude of the observed performance responses, establish optimal inclusion strategies, and evaluate the role of WF-32016 within integrated *Salmonella* control programs.

## Author contribution

**Lydia Zeibich** made substantial contributions to the conception and design of the work, interpretation of the data, presentation of the results, drafting of the manuscript, and critical review and revision of the manuscript for crucial intellectual content. **Alexandra Schlagheck** made substantial contributions to the conception and design of the work and to the critical review and revision of the manuscript for crucial intellectual content. **Ali R. Al Sulaiman** made substantial contributions to conducting the experimental procedures, acquisition, organization, and interpretation of the data, presentation of the results, and critical review and revision of the manuscript for crucial intellectual content. **Max Ingberman** made substantial contributions to the conception and design of the work, implementation of the experimental procedures, organization, validation, and analysis of the data, and critical review and revision of the manuscript for crucial intellectual content. **Ala E. Abudabos** made substantial contributions to the conception of the work, validation and analysis of the data, provision of research resources, scientific supervision and coordination of the research, securing financial support for the work, and critical review and revision of the manuscript for crucial intellectual content. **Breno Castello Branco Beirão** made substantial contributions to the conception of the work, analysis and interpretation of the data, provision of research resources, scientific supervision and coordination of the research, securing financial support for the work, and critical review and revision of the manuscript for crucial intellectual content. All authors reviewed and explicitly approved the exact final version of the manuscript and agreed to be personally and publicly accountable for all aspects of the work, ensuring that any questions related to the accuracy or integrity of the research are thoroughly investigated and resolved.

## Disclosures

The authors declare that they have no conflict of interest.

## Footnotes

Supplementary material associated with this article can be found, in the online version, at doi:10.1016/j.psj.2026.107881.

## References

[bib0002] Abdel-Raheem S.M., Abd El-Hamid M.I., Khamis T., Baz H.A., Omar A.E., Gad W.M., El-Azzouny M.M., Habaka M.A.M., Mohamed R.I., Elkenawy M.E., Dawod R.E., Elalfy E.A., Ibrahim D. (2024). Comprehensive efficacy of nano-formulated mixed probiotics on broiler chickens’ performance and *Salmonella* Typhimurium challenge. Poult. Sci..

[bib0001] Abdelqader A., Al-Fataftah A.R. (2016). Effect of dietary butyric acid on performance, intestinal morphology, microflora composition and intestinal recovery of heat-stressed broilers. Livest. Sci..

[bib0003] Alikhan N.F., Moreno L.Z., Castellanos L.R., Chattaway M.A., McLauchlin J., Lodge M., OGrady J., Zamudio R., Doughty E., Petrovska L., Cunha M.P.V., Knöbl T., Moreno A.M., Mather A.E. (2022). Dynamics of *Salmonella* enterica and antimicrobial resistance in the Brazilian poultry industry and global impacts on public health. PLoS Genet..

[bib0004] AOAC (2019).

[bib0005] Aviagen (2022).

[bib0006] Bampidis V., Azimonti G., de L. Bastos M., Christensen H., Dusemund B., Kouba M., Kos Durjava M., López-Alonso M., López Puente S., Marcon F., Mayo B., Pechová A., Petkova M., Ramos F., Sanz Y., Villa R.E., Woutersen R., Dierick N., Martelli G., Cocconcelli P.S., Glandorf B., Herman L., Prieto Maradona M., Saarela M., Anguita M., Brozzi R. (2020). Safety and efficacy of TechnoSpore® (*Bacillus coagulans* DSM 32016) for piglets, other growing Suidae, chickens for fattening, other poultry for fattening and ornamental birds. EFSA J..

[bib0007] Blodgett R. (2023).

[bib0008] Bourriaud C., Robins R.J., Martin L., Kozlowski F., Tenailleau E., Cherbut C., Michel C. (2005). Lactate is mainly fermented to butyrate by human intestinal microfloras but inter-individual variation is evident. J. Appl. Microbiol..

[bib0009] Cao J., Yu Z., Liu W., Zhao J., Zhang H., Zhai Q., Chen W. (2020). Probiotic characteristics of Bacillus coagulans and associated implications for human health and diseases. J. Funct. Foods.

[bib0010] Chattaway M.A., Langridge G.C., Wain J. (2021). *Salmonella* nomenclature in the genomic era: a time for change. Sci. Rep..

[bib0011] Chen H., Liu W., Zhang H., Yan Y., Chen M., Ding X., Zhang C., Jiang R., Wang Z. (2024). Effects of Bacillus subtilis KG109 on growth performance, carcass quality, serum indicators, intestinal morphology, and digestive enzymes in broilers. Animals.

[bib0012] Chung K.C., Goepfert J.M. (1970). Growth of *salmonella* at low pH. J. Food Sci..

[bib0013] Dar M.A., Ahmad S.M., Bhat S.A., Ahmed R., Urwat U., Mumtaz P.T., Bhat S.A., Dar T.A., Shah R.A., Ganai N.A. (2017). *Salmonella* typhimurium in poultry: a review. Worlds Poult. Sci. J..

[bib0014] Dolin B.J. (2009). Effects of a proprietary Bacillus coagulans preparation on symptoms of diarrhea-predominant irritable bowel syndrome. Methods Find. Exp. Clin. Pharmacol..

[bib0015] dos Santos A.M.P., Panzenhagen P., Ferrari R.G., Portes A.B., de Jesus A.C., Ochioni A., Rodrigues D., Toro M., Meng J., Allard M., Conte-Junior C.A. (2024). Genomic characterization of a clonal emergent *Salmonella* Minnesota lineage in Brazil reveals the presence of a novel megaplasmid of resistance and virulence. Appl. Environ. Microbiol..

[bib0016] Duncan S.H., Louis P., Flint H.J. (2004). Lactate-utilizing bacteria, isolated from human feces, that produce butyrate as a major fermentation product. Appl. Environ. Microbiol..

[bib0017] El Mahdy A.M., Badr J.M., Mansour M.K. (2021). The effect of probiotic and colistin sulphate on infection with *Salmonella Enteritidis* in the broilers. World J. Pharm. Pharm. Sci..

[bib0018] Elleithy E.M.M., Bawish B.M., Kamel S., Ismael E., Bashir D.W., Hamza D., din Fahmy K.N.E. (2023). Influence of dietary *Bacillus coagulans* and/or *Bacillus licheniformis*-based probiotics on performance, gut health, gene expression, and litter quality of broiler chickens. Trop. Anim. Health Prod..

[bib0019] FAO/WHO (2001). Report of a Joint FAO/WHO Expert Consultation on Evaluation of Health and Nutritional Properties of Probiotics in Food Including Powder Milk with Live Lactic Acid Bacteria.

[bib0020] Gao G., Zhou J., Wang H., Ding Y., Zhou J., Chong P.H., Zhu L., Ke L., Wang X., Rao P., Wang Q., Zhang L. (2022). Effects of valerate on intestinal barrier function in cultured Caco-2 epithelial cell monolayers. Mol. Biol. Rep..

[bib0021] Garrote G.L., Abraham A.G., Rumbo M. (2015). Is lactate an undervalued functional component of fermented food products?. Front. Microbiol..

[bib0022] Govoni C., Chiarelli D.D., Luciano A., Ottoboni M., Perpelek S.N., Pinotti L., Rulli M.C. (2021). Global assessment of natural resources for chicken production. Adv. Water Resour..

[bib0023] Grimont P.A.D., Weill F.-X. (2007). WHO Collaborating Centre for Reference and Research on *Salmonella*.

[bib0024] Hashemitabar S.H., Hosseinian S.A. (2024). The comparative effects of probiotics on growth, antioxidant indices and intestinal histomorphology of broilers under heat stress condition. Sci. Rep..

[bib0025] Idowu P.A., Mpofu T.J., Magoro A.M., Modiba M.C., Nephawe K.A., Mtileni B. (2025). Impact of probiotics on chicken gut microbiota, immunity, behavior, and productive performance—a systematic review. Front. Anim. Sci..

[bib0026] Khasanah H., Kusbianto D.E., Purnamasari L., Dela Cruz J.F., Widianingrum D.C., Hwang S.G. (2024). Modulation of chicken gut microbiota for enhanced productivity and health: a review. Vet. World.

[bib0027] Khurajog B., Saenkankam I., Apiwatsiri P., Supimon N., Kamwa R., Niyomtham W., Yindee J., Phupolphan C., Hampson D.J., Prapasarakul N. (2026). Effectiveness of probiotic supplementation on growth performance, gut microbiota, and *Salmonella* reduction in broiler chicks challenged with *Salmonella* Typhimurium. Sci. Rep..

[bib0028] Kim H., Lee J., Kim S., Kim B., Chang S., Song D., Park S., Jeon K., Cho J. (2025). Supplemental illite or in combination with probiotics improves performance and intestinal health in broiler chickens challenged with *Salmonella* Typhimurium. J. Anim. Sci. Technol..

[bib0029] Lee M.D., Pedroso A.A., Lumpkins B., Cho Y., Maurer J.J. (2023). Pioneer colonizers: bacteria that alter the chicken intestinal morphology and development of the microbiota. Front. Physiol..

[bib0030] Liu Y., Li Z., Li H., Wan S., Tang S. (2023). Bacillus pumilus TS1 alleviates *Salmonella* Enteritidis-induced intestinal injury in broilers. BMC Vet. Res..

[bib0031] Lou H., Wang J., Wang Y., Gao Y., Wang W. (2024). Protective effects of potential probiotics lacticaseibacillus rhamnosus SN21-1 and lactiplantibacillus plantarum SN21-2 against *Salmonella* typhimurium infection in broilers. Poult. Sci..

[bib0032] Marcato F., Schokker D., Kar S.K., Bossers A., Harders F., Rebel J.M.J., Jansen C.A., van der Valk E., Kruijt L., te Beest D.E., de Jong I.C. (2024). Effects of breed and early feeding on intestinal microbiota, inflammation markers, and behavior of broiler chickens. Front. Vet. Sci..

[bib0033] Maurer J.J., Cheng Y., Pedroso A., Thompson K.K., Akter S., Kwan T., Morota G., Kinstler S., Porwollik S., McClelland M., Escalante-Semerena J.C., Lee M.D. (2024). Peeling back the many layers of competitive exclusion. Front. Microbiol..

[bib0034] McLendon B.L., Cox N.A., Cosby D.E., Montiel E.R., Russell S.M., Hofacre C.L., Berrang M.E., Wilson J.L. (2020). Detection of *salmonella* in young chicks with cloacal swabs. Adv. Food Nutr. Sci..

[bib0035] Naeem M., Bourassa D. (2024). Optimizing poultry nutrition to combat *Salmonella*: insights from the literature. Microorganisms.

[bib0036] Nair D.V.T., Kollanoor Johny A. (2019). Food Safety in Poultry Meat Production.

[bib0037] Nazari Moghadam M., Rahimi E., Shakerian A., Momtaz H. (2023). Prevalence of *Salmonella* Typhimurium and *Salmonella* Enteritidis isolated from poultry meat: virulence and antimicrobial-resistant genes. BMC Microbiol..

[bib0038] Parhizkar S., Zaghari M., Zhandi M. (2022). Beneficial effect of *Bacillus coagulans* DSM 32016 on performance and productivity of broiler breeders. J. Livest. Sci. Technol..

[bib0039] Payot T., Chemaly Z., Fick M. (1999). Lactic acid production by Bacillus coagulans—kinetic studies and optimization of culture medium for batch and continuous fermentations. Enzyme Microb. Technol..

[bib0040] Queiroz A.C., Milanesi M., Queiroz L.H., Nunes C.M. (2023). Occurrence of *Salmonella* spp. in the broiler production chain. Arq. Bras. Med. Vet. Zootec..

[bib0041] Raheem A., Liang L., Zhang G., Cui S. (2021). Modulatory effects of probiotics during pathogenic infections with emphasis on immune regulation. Front. Immunol..

[bib0042] Shanmugasundaram R., Khochamit N., Selvaraj R.K., Mortada M., Siripornadulsil S., Siripornadulsil W. (2025). In vitro characterization of probiotic strains Bacillus subtilis and Enterococcus durans and their effect on broiler chicken performance and immune response during *Salmonella* Enteritidis infection. Microorganisms.

[bib0043] Sinnelä M.T., Pawluk A.M., Jin Y.H., Kim D., Mah J.H. (2021). Effect of calcium and manganese supplementation on heat resistance of spores of Bacillus species associated with food poisoning, spoilage, and fermentation. Front. Microbiol..

[bib0044] Tellez G., Pixley C., Wolfenden R.E., Layton S.L., Hargis B.M. (2012). Probiotics/direct fed microbials for *Salmonella* control in poultry. Food Res. Int..

[bib0045] Thames H.T., Fancher C.A., Colvin M.G., McAnally M., Tucker E., Zhang L., Kiess A.S., Dinh T.T.N., Sukumaran A.T. (2022). The prevalence of *Salmonella* and Campylobacter on broiler meat at different stages of commercial poultry processing. Animals.

[bib0046] Tykałowski B., Koncicki A. (2024). National and international trade in poultry and poultry products in light of the valid legal regulations. Med. Weter..

[bib0047] Van Immerseel F., Russell J.B., Flythe M.D., Gantois I., Timbermont L., Pasmans F., Haesebrouck F., Ducatelle R. (2006). The use of organic acids to combat *Salmonella* in poultry: a mechanistic explanation of the efficacy. Avian Pathol..

[bib0048] Vicuña E.A., Kuttappan V.A., Tellez G., Hernandez-Velasco X., Seeber-Galarza R., Latorre J.D., Faulkner O.B., Wolfenden A.D., Hargis B.M., Bielke L.R. (2015). Dose titration of FITC-D for optimal measurement of enteric inflammation in broiler chicks. Poult. Sci..

[bib0049] Wang J., Fenster D.A., Vaddu S., Bhumanapalli S., Kataria J., Sidhu G., Leone C., Singh M., Dalloul R.A., Thippareddi H. (2024). Colonization, spread and persistence of *Salmonella* (Typhimurium, Infantis and Reading) in internal organs of broilers. Poult. Sci..

[bib0050] Wu Y., Shao Y., Song B., Zhen W., Wang Z., Guo Y., Shahid M.S., Nie W. (2018). Effects of *Bacillus coagulans* supplementation on the growth performance and gut health of broiler chickens with Clostridium perfringens-induced necrotic enteritis. J. Animal Sci. Biotechnol..

[bib0051] Yin Y., Peng H., Bai H., Pei Z., Chen Z., Ma C., Zhu M., Li J., Li C., Gong Y., Wang L., Teng L., Qin Z., Zhou J., Wei T., Liao Y. (2024). Enhancing resistance to *Salmonella* Typhimurium in yellow-feathered broilers: a study of a strain of *lactiplantibacillus plantarum* as probiotic feed additives. Front. Microbiol..

[bib0052] Zhang S., Li P., Lee S., Wang Y., Tan C., Shang N. (2024). *Weizmannia coagulans*: an ideal probiotic for gut health. Food Sci. Hum. Wellness.

[bib0053] Zhang Y., Zhou J., Ji L., Zhang L., Zhao L., Guo Y., Wei H., Lu L. (2024). *Bacillus subtilis* improves antioxidant capacity and optimizes inflammatory state in broilers. Anim. Biosci..

[bib0054] Zhen W., Shao Y., Gong X., Wu Y., Geng Y., Wang Z., Guo Y. (2018). Effect of dietary *Bacillus coagulans* supplementation on growth performance and immune responses of broiler chickens challenged by *Salmonella enteritidis*. Poult. Sci..

[bib0055] Zhou Y., Zeng Z., Xu Y., Ying J., Wang B., Majeed M., Majeed S., Pande A., Li W. (2020). Application of Bacillus coagulans in animal husbandry and its underlying mechanisms. Animals.

[bib0056] Zizza A., Fallucca A., Guido M., Restivo V., Roveta M., Trucchi C. (2024). Foodborne infections and *Salmonella*: current primary prevention tools and future perspectives. Vaccines (Basel).

