# Supplementary material for: Dietary Weizmannia faecalis DSM 32016 probiotic supplementation reduces Salmonella colonization and supports growth performance in broilers challenged with Salmonella enterica serovars
Source: Poult Sci. 2026 Oct 5;105(12):107881. doi: 10.1016/j.psj.2026.107881 (PMC13677393; doi:10.1016/j.psj.2026.107881)
Supplement: Supplementary file 1 [file mmc1.docx]

**Table S1.** Multivariate analysis of the effects of probiotic supplementation, *Salmonella* Typhimurium challenge, and their interaction on growth performance parameters in Study I.

| **Source of variation** | **Starter phase** | **Finisher phase** | **Overall period** |
| --- | --- | --- | --- |
| Daily feed intake |  |  |  |
| *Salmonella* challenge | 0.015 | 0.894 | 0.247 |
| Probiotic starter-phase supplementation | 0.698 | 0.159 | 0.416 |
| *Salmonella* × probiotic starter phase | 0.368 | 0.985 | 0.719 |
| Probiotic finisher-phase supplementation | N.A. | 0.343 | 0.642 |
| *Salmonella* × probiotic finisher phase | N.A. | 0.974 | 0.938 |
| Daily weight gain |  |  |  |
| *Salmonella* challenge | 0.006 | 0.002 | 0.002 |
| Probiotic starter-phase supplementation | 0.868 | <0.001 | 0.021 |
| *Salmonella* × probiotic starter phase | 0.217 | 0.237 | 0.095 |
| Probiotic finisher-phase supplementation | N.A. | <0.001 | 0.021 |
| *Salmonella* × probiotic finisher phase | N.A. | 0.402 | 0.207 |
| Feed conversion ration |  |  |  |
| *Salmonella* challenge | 0.034 | <0.001 | <0.001 |
| Probiotic starter-phase supplementation | 0.460 | <0.001 | 0.002 |
| *Salmonella* × probiotic starter phase | 0.270 | 0.017 | 0.024 |
| Probiotic finisher-phase supplementation | N.A. | <0.001 | 0.002 |
| *Salmonella* × probiotic finisher phase | N.A. | 0.028 | 0.051 |
| Production efficiency factor |  |  |  |
| *Salmonella* challenge | 0.005 | <0.001 | <0.001 |
| Probiotic starter-phase supplementation | 0.930 | <0.001 | 0.011 |
| *Salmonella* × probiotic starter phase | 0.237 | 0.033 | 0.051 |
| Probiotic finisher-phase supplementation | N.A. | <0.001 | 0.017 |
| *Salmonella* × probiotic finisher phase | N.A. | 0.082 | 0.140 |

Values are *P* values from multivariate analysis of variance (MANOVA). Statistical significance was set at *P* < 0.05. N.A., not applicable.
